# Ambient air pollution contributed to pulmonary tuberculosis in China

**DOI:** 10.1080/22221751.2024.2399275

**Published:** 2024-08-29

**Authors:** Zhongqi Li, Qiao Liu, Liang Chen, Liping Zhou, Wei Qi, Chaocai Wang, Yu Zhang, Bilin Tao, Limei Zhu, Leonardo Martinez, Wei Lu, Jianming Wang

**Affiliations:** a Department of Epidemiology, Center for Global Health, School of Public Health, Nanjing Medical University, Nanjing, People’s Republic of China; b Department of Chronic Communicable Disease, Center for Disease Control and Prevention of Jiangsu Province, Nanjing, People’s Republic of China; c Guangdong Provincial Institute of Public Health, Guangzhou, People’s Republic of China; d Institute of Tuberculosis Control, Center for Disease Control and Prevention of Hubei Province, Wuhan, People’s Republic of China; e Department of tuberculosis, Center for Disease Control and Prevention of Liaoning Province, Shenyang, People’s Republic of China; f Department of tuberculosis, Center for Disease Control and Prevention of Qinghai Province, Xining, People’s Republic of China; g Department of Epidemiology, School of Public Health, Boston University, Boston, MA, USA

**Keywords:** Outdoor air pollutants, pulmonary tuberculosis, time-series, risk

## Abstract

Published studies on outdoor air pollution and tuberculosis risk have shown heterogeneous results. Discrepancies in prior studies may be partially explained by the limited geographic scope, diverse exposure times, and heterogeneous statistical methods. Thus, we conducted a multi-province, multi-city time-series study to comprehensively investigate this issue. We selected 67 districts or counties from all geographic regions of China as study sites. We extracted data on newly diagnosed pulmonary tuberculosis (PTB) cases, outdoor air pollutant concentrations, and meteorological factors in 67 sites from January 1, 2014 to December 31, 2019. We utilized a generalized additive model to evaluate the relationship between ambient air pollutants and PTB risk. Between 2014 and 2019, there were 172,160 newly diagnosed PTB cases reported in 67 sites. With every 10-μg/m^3^ increase in SO_2_, NO_2_, PM_10_, PM_2.5_, and 1-mg/m^3^ in CO, the PTB risk increased by 1.97% [lag 0 week, 95% confidence interval (CI): 1.26, 2.68], 1.30% (lag 0 week, 95% CI: 0.43, 2.19), 0.55% (lag 8 weeks, 95% CI: 0.24, 0.85), 0.59% (lag 10 weeks, 95% CI: 0.16, 1.03), and 5.80% (lag 15 weeks, 95% CI: 2.96, 8.72), respectively. Our results indicated that ambient air pollutants were positively correlated with PTB risk, suggesting that decreasing outdoor air pollutant concentrations may help to reduce the burden of tuberculosis in countries with a high burden of tuberculosis and air pollution.

## Introduction

Globally, tuberculosis is one of the leading causes of mortality, and pulmonary tuberculosis (PTB) accounts for the vast majority of these deaths. Approximately 10.6 million new tuberculosis cases and 1.6 million tuberculosis-related deaths were documented worldwide in 2021 [1]. An estimated quarter of the global population is infected with *Mycobacterium tuberculosis* (*M.tb*), namely, latent tuberculosis infection. Risk factors for infection and the development of active tuberculosis include smoking and diabetes [1]. Recently, some studies have reported that outdoor air pollutants contribute to tuberculosis risk [2,3].

Ambient air pollution has posed a sizable threat to public health. Millions of deaths worldwide are directly attributed to outdoor air pollution each year [4]. A global analysis revealed that PM_10_ and PM_2.5_ were positively associated with all-cause, cardiovascular, and respiratory mortality [5]. Numerous studies have shown that outdoor air pollution probably contributes to various diseases, such as ischemic stroke [6], asthma [7], and childhood pneumonia [8,9].

Exposure to diesel exhaust may reduce the expression of several cytokines, such as tumour necrosis factor-α (TNF-α) and interferon-γ (IFN-γ) in murine lung tissues, which play critical roles in host defense [10,11]. Diesel exhaust contains a variety of air pollutants, including SO_2_, NO_2_, particulate matter, and CO. This has led to the hypothesis that outdoor air pollution exposure may increase lung infection acquisition. Although some studies have explored the role of outdoor air pollution in the tuberculosis risk, the results have been heterogeneous. In Chengdu, SO_2_, NO_2_, and PM_10_ were positively related to tuberculosis [3]. Another study from northern California suggested that PTB was positively associated with NO_2_ and CO and negatively associated with PM_10_ and O_3_ but not significantly associated with SO_2_ and PM_2.5_ [2]. In Seoul, SO_2_ was shown to be positively linked to tuberculosis [12]. A study from Jinan revealed that PTB was negatively correlated with NO_2_ and positively correlated with O_3_ [13].

Inconsistent results may be due to the fact that these studies were conducted in a sole region or city, explored diverse exposure times, and applied distinct statistical methods. To address this knowledge gap, we performed a large, multi-province study in 67 study sites throughout China to investigate the impact of outdoor air pollution on the risk of PTB.

## Materials and methods

### Study areas

Currently, China has 34 provincial administrative divisions. We selected Jiangsu Province, Guangdong Province, Qinghai Province, Liaoning Province, and Hubei Province as the study areas for eastern, southern, western, northern, and central China, respectively. According to geographical location, history, culture and nationality, these 34 provincial administrative regions can be divided into seven regions, namely, eastern, southern, southwestern, northwestern, northern, northeastern, and central China. First, we combined these seven regions into five regions, namely, eastern, southern, western (southwestern and northwestern), northern (northern and northeastern), and central China. Each region contained several provincial administrative divisions. Subsequently, we selected two or three provincial administrative divisions from each region as alternative study areas after comprehensively considering the average annual reported PTB incidence (low incidence: < 50/100,000; middle incidence: ≥ 50/100,000 and ≤100/100,000; high incidence: > 100/100,000) from 2014 to 2019 and the basis of previous cooperation. Then, we contacted the heads of the TB prevention and control institutions in these candidate study areas to ask whether they were interested in cooperating to perform this study. Jiangsu Province (with a low incidence), Guangdong Province (with a moderate incidence), Qinghai Province (with a high incidence), Liaoning Province (with a moderate incidence), and Hubei Province (with a moderate incidence) expressed their willingness to participate in this study.

These five provinces contain 69 municipal administrative divisions. We chose one county-level administrative division in each municipal administrative division. The following criteria were used: (1) availability of data on environmental factors; (2) no change in the administrative division between 2014 and 2019; and (3) the grade of environmental monitoring stations (prioritized order of air pollutant monitoring stations: national control stations > provincial control stations > municipal control stations > county control stations; prioritized order of meteorological factor monitoring stations: national benchmark climate stations > national basic meteorological stations > national general meteorological stations). After excluding two municipal administrative divisions without county-level administrative divisions, a total of 67 county-level administrative divisions were identified as study sites.

### Data collection

We collected daily data on newly diagnosed PTB patients between January 1, 2014, and December 31, 2019, at 67 study sites, including sex, age, occupation, and diagnosis date, from the China Tuberculosis Management Information System. Sensitive and identifiable information such as names, phone numbers, and address details of the cases were removed to protect confidentiality and privacy. From this time period, daily data on outdoor air pollutant concentrations and meteorological factors from these sites were extracted from the National Urban Air Quality Real-time Release Platform and the China Meteorological Data Sharing Center, respectively, which were widely utilized in previous studies [14,15]. The outdoor air pollutants included SO_2_ (μg/m^3^), NO_2_ (μg/m^3^), PM_10_ (μg/m^3^), PM_2.5_ (μg/m^3^), and CO (mg/m^3^). The average meteorological factors included temperature (℃), wind speed (m/s), and relative humidity (%). We calculated the weekly number of PTB cases, the mean concentration of each air pollutant weekly, and the mean value of each meteorological factor weekly. The study period was then divided into 313 weeks.

### Statistical analysis

We applied a generalized additive model (GAM) following the quasi-Poisson distribution to evaluate the relationship between outdoor air pollutant concentrations and the PTB risk [16,17]. According to previous studies, outdoor air pollutants may have lag effects on tuberculosis risk [3,18]. Thus, we determined the maximum lag time up to 25 weeks to capture both short-term (lag 0 week to lag 4 weeks) and relatively long-term (lag 5 weeks to lag 25 weeks) impacts of outdoor air pollutants [17,19]. For example, SO_2_ at lag 0 week indicated the weekly average concentration of SO_2_ at the current week, and SO_2_ at lag 1 week indicated the weekly average concentration of SO_2_ one week prior. Covariates adjusted in the model included “week” (week  = 1, 2, … , 313), the number of cases one week ago, the number of holidays in the week, season, “city” to control city-specific characteristics such as socioeconomic level and population scope, and three weekly average meteorological factors at the same lag week [17,20,21]. We created smooth terms for “week” and meteorological factors using the thin plate spline function (TPSF) with maximum degrees of freedom (*df*) of six and two, respectively, to control their potential nonlinear effects [17,22].

We calculated the influences of ambient air pollutants on the PTB risk at all lag weeks and then identified the lag week with the maximum effect (air pollutant concentration and the PTB risk showed the strongest association at this lag week) for each air pollutant to perform further analyses. The strength of the relationship is presented as percentage changes in the PTB risk and the 95% confidence intervals (CIs) for each 10-μg/m^3^ (1-mg/m^3^ for CO) increase in air pollutant concentration. We conducted subgroup analyses to estimate the associations between air pollutants and the PTB risk in different populations and seasons. The differences in effects between subgroups were evaluated using the formula: $|{\text{β}}_{1}-{\text{β}}_{2}|/\sqrt{SE_{1}^{2}+SE_{2}^{2}},$ where ${\text{β}}_{1}$ and ${\text{β}}_{2}$ are the estimated effects and $SE_{1}$ and $SE_{2}$ are the standard errors of the estimates. The difference between different sex groups or age groups was considered to be statistically significant if the corresponding value was >1.96, and the difference between different season groups was considered to be statistically significant after the Bonferroni correction if the corresponding value was >2.64 [23].

We further performed a series of sensitivity analyses to assess the stability of the relationships between air pollutants and the PTB risk. First, we constructed dual-pollutant models by additionally adjusting each of the other air pollutants in the model. Notably, due to the high correlation between PM_10_ and PM_2.5_ (Figure S1), they were not entered into the model simultaneously to address the multicollinearity problem [5,20]. Second, we varied the maximum *df* of the smooth term of “week” between four and eight and the maximum *df* of the smooth terms of meteorological factors between two and six to investigate the impacts of air pollutants. Third, since monitoring data on ambient air pollutants in some study sites started on December 31, 2014 (week 53), we recalculated the effects at 53–313 weeks. Fourth, we considered “city” as the random effect variable and re-evaluated the associations between air pollutant concentrations and the PTB risk using the generalized additive mixed model (GAMM). GAMM is an extension of the GAM that adds a random effect term on the basis of the GAM [24,25].

Moreover, considering our assumption that the links between air pollutants and the PTB risk were linear, we plotted the concentration-response curve between each air pollutant and the PTB risk to examine the reliability of our results. Specifically, we constructed a smooth term for each air pollutant using the TPSF with a maximum *df* of two to replace the linear term in the model. We applied the piecewise linear function to re-estimate the effect if the curve was nonlinear [17]. The optimal cutoff value of the piecewise linear function was determined by the minimum generalized cross-validation score [5].

All analyses were conducted in R software, and the significance level was set as 0.05 (2-tailed).

## Results

### Characteristics of PTB patients at 67 sites

A total of 172,160 patients were newly diagnosed with PTB at 67 sites between 2014 and 2019. Among them, 122,074 (70.91%) were males, the mean age was 47.59 ± 18.98 years, 79,895 (46.41%) were farmers or workers, 92,083 (53.49%) were from southern China, and 30,352 (17.63%) were reported in 2014 (Table 1).

**Table 1.** Characteristics of pulmonary tuberculosis patients at the 67 study sites.

Characteristics	Statistics
Sex [n (%)]	
Male	122074 (70.91)
Female	50086 (29.09)
Age (years, mean ± SD)	47.59 ± 18.98
Occupation [n (%)]	
Students	7102 (4.12)
Farmers or workers	79895 (46.41)
Others	85163 (49.47)
Region [n (%)]	
Eastern China	20472 (11.89)
Southern China	92083 (53.49)
Western China	8652 (5.03)
Northern China	21077 (12.24)
Central China	29876 (17.35)
Year [n (%)]	
2014	30352 (17.63)
2015	29466 (17.11)
2016	28314 (16.45)
2017	29708 (17.26)
2018	28015 (16.27)
2019	26305 (15.28)

Abbreviation: SD = standard deviation.

### Description of the outdoor air pollutants at the 67 sites

Between 2014 and 2019, the median (Q25, Q75) weekly average SO_2_, NO_2_, PM_10_, PM_2.5_, and CO concentrations at the 67 sites were 13.57 μg/m^3^ (9.00, 21.29), 27.43 μg/m^3^ (18.71, 38.00), 63.43 μg/m^3^ (44.57, 90.00), 36.86 μg/m^3^ (25.43, 53.57), and 0.90 mg/m^3^ (0.73, 1.13), respectively (Table S1).

### Outdoor air pollutants and PTB risk

**Figure 1.**
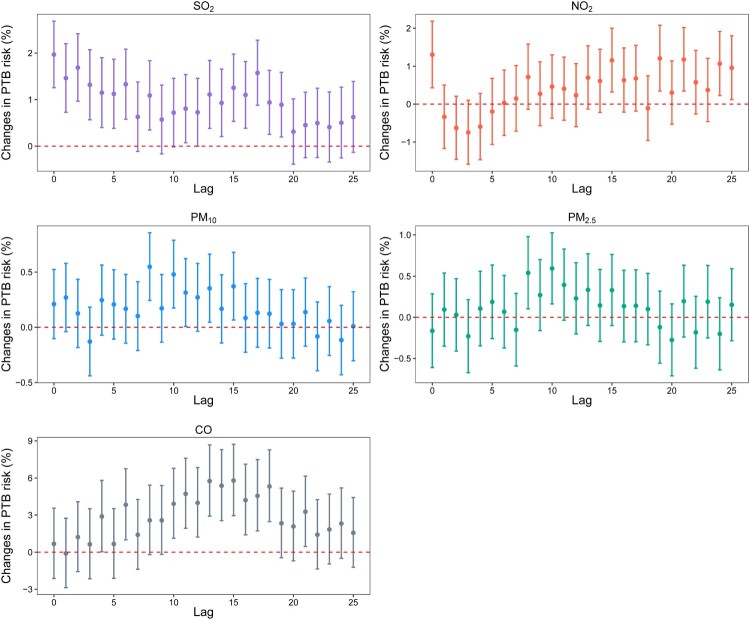
Percentage changes in pulmonary tuberculosis risk and their 95% confidence intervals for each 10-μg/m^3^ (1-mg/m^3^ for CO) increase in ambient air pollutant concentrations at all lag weeks. Abbreviation: PTB = pulmonary tuberculosis. The results were adjusted for the week, the number of cases in the previous week, the number of holidays in the week, season, city, average temperature, average wind speed, and average relative humidity at the same lag week.

Outdoor air pollutants were significantly associated with the PTB risk at multiple lag weeks, including lag 0 week to lag 6 weeks, lag 8 weeks, and lag 11 weeks to lag 19 weeks for SO_2_; lag 0 week, lag 15 weeks, lag 19 weeks, lag 21 weeks, lag 24 weeks, and lag 25 weeks for NO_2_; lag 8 weeks, lag 10 weeks, lag 11 weeks, lag 13 weeks, and lag 15 weeks for PM_10_; lag 8 weeks and lag 10 weeks for PM_2.5_; lag 4 weeks, lag 6 weeks, lag 10 weeks to lag 18 weeks, and lag 21 weeks for CO (Figure 1).

The strongest associations between air pollutants and the PTB risk appeared at lag 0 week for SO_2_ and NO_2_, lag 8 weeks for PM_10_, lag 10 weeks for PM_2.5_, and lag 15 weeks for CO. At the corresponding lag weeks, for every 10-μg/m^3^ increase in SO_2_, NO_2_, PM_10_, PM_2.5_, and 1-mg/m^3^ in CO, the PTB risk increased by 1.97% (95% CI: 1.26, 2.68), 1.30% (95% CI: 0.43, 2.19), 0.55% (95% CI: 0.24, 0.85), 0.59% (95% CI: 0.16, 1.03), and 5.80% (95% CI: 2.96, 8.72), respectively (Table 2).

**Table 2.** Percentage changes in pulmonary tuberculosis risk and their 95% confidence intervals for each 10-μg/m^3^ (1-mg/m^3^ for CO) increase in ambient air pollutant concentrations*.

Model	SO_2_	NO_2_	PM_10_	PM_2.5_	CO
Model 1^†^	1.97 (1.26, 2.68)	1.30 (0.43, 2.19)	0.55 (0.24, 0.85)	0.59 (0.16, 1.03)	5.80 (2.96, 8.72)
Model 2^‡^	NA	0.57 (−0.29, 1.45)	0.45 (0.13, 0.78)	0.51 (0.06, 0.97)	4.78 (1.83, 7.81)
Model 3^§^	1.83 (1.09, 2.58)	NA	0.55 (0.21, 0.91)	0.62 (0.13, 1.11)	5.06 (2.07, 8.14)
Model 4^ll^	2.00 (1.27, 2.74)	1.31 (0.31, 2.33)	NA	NA	5.25 (2.21, 8.39)
Model 5**	2.19 (1.46, 2.93)	1.84 (0.85, 2.84)	NA	NA	5.80 (2.75, 8.95)
Model 6^††^	2.07 (1.34, 2.82)	1.36 (0.44, 2.29)	0.51 (0.19, 0.84)	0.42 (−0.04, 0.89)	NA

Abbreviation: NA = not available. *: We applied lag 0 week for SO_2_, lag 0 week for NO_2_, lag 8 weeks for PM_10_, lag 10 weeks for PM_2.5_, and lag 15 weeks for CO. ^†^: Adjusted for the week, the number of cases in the previous week, the number of holidays in the week, season, city, average temperature, average wind speed, and average relative humidity at the same lag week. ^‡^: Based on Model 1, additionally adjusted for SO_2_ at the same lag week. ^§^: Based on Model 1, additionally adjusted for NO_2_ at the same lag week. ^ll^: Based on Model 1, additionally adjusted for PM_10_ at the same lag week. **: Based on Model 1, additionally adjusted for PM_2.5_ at the same lag week. ^††^: Based on Model 1, additionally adjusted for CO at the same lag week.

The subgroup analyses indicated that the associations between air pollutants and the PTB risk were significant in males, females, people aged <60 years, people aged ≥60 years, autumn, and winter for SO_2_; in males, females, people <60 years old, and winter for NO_2_; in males, people aged <60 years, people aged ≥60 years, and winter for PM_10_; in males, people <60 years old, and winter for PM_2.5_; and in males, females, people aged <60 years, people aged ≥60 years, summer, and winter for CO (Table 3). Moreover, the differences in the associations between air pollutants and the PTB risk were not statistically significant between different sex groups or age groups but were statistically significant between spring and winter for NO_2_, PM_10_, and PM_2.5_ (Table S2).

**Table 3.** Subgroup analyses of percentage changes in pulmonary tuberculosis risk and their 95% confidence intervals for each 10-μg/m^3^ (1-mg/m^3^ for CO) increase in ambient air pollutant concentrations*.

Variables	SO_2_	NO_2_	PM_10_	PM_2.5_	CO
Sex^†^					
Male	1.98 (1.20, 2.76)	1.34 (0.38, 2.31)	0.59 (0.25, 0.93)	0.71 (0.24, 1.19)	5.30 (2.14, 8.55)
Female	2.13 (0.97, 3.31)	1.94 (0.59, 3.32)	0.34 (−0.12, 0.81)	0.21 (−0.46, 0.89)	7.88 (3.60, 12.34)
Age group^†^					
< 60 years old	2.08 (1.26, 2.90)	1.59 (0.61, 2.58)	0.50 (0.16, 0.84)	0.58 (0.10, 1.07)	5.90 (2.76, 9.13)
≥ 60 years old	2.08 (0.92, 3.26)	1.03 (−0.25, 2.32)	0.55 (0.08, 1.02)	0.63 (−0.03, 1.28)	6.72 (2.27, 11.37)
Season^‡^					
Spring	0.68 (−1.25, 2.65)	−0.12 (−1.96, 1.75)	−0.20 (−0.68, 0.28)	−0.03 (−0.67, 0.60)	4.37 (−0.13, 9.06)
Summer	2.54 (−0.98, 6.18)	0.56 (−2.05, 3.24)	0.52 (−0.21, 1.25)	0.37 (−0.88, 1.65)	7.75 (2.16, 13.66)
Autumn	2.33 (0.45, 4.24)	0.07 (−1.82, 1.99)	0.38 (−0.67, 1.44)	0.58 (−0.93, 2.12)	−0.04 (−7.22, 7.70)
Winter	2.68 (1.41, 3.96)	3.29 (1.66, 4.94)	1.30 (0.66, 1.95)	1.52 (0.57, 2.48)	9.82 (1.85, 18.41)

*: We applied lag 0 week for SO_2_, lag 0 week for NO_2_, lag 8 weeks for PM_10_, lag 10 weeks for PM_2.5_, and lag 15 weeks for CO. ^†^: Adjusted for the week, the number of cases in the previous week, the number of holidays in the week, season, city, average temperature, average wind speed, and average relative humidity at the same lag week. ^‡^: Adjusted for the week, the number of cases in the previous week, the number of holidays in the week, city, average temperature, average wind speed, and average relative humidity at the same lag week.

The sensitivity analyses showed that the associations between air pollutants and the PTB risk remained significant after adjusting for NO_2_, PM_10_, PM_2.5_, or CO for SO_2_; after adjusting for PM_10_, PM_2.5_, or CO for NO_2_; after adjusting for SO_2_, NO_2_, or CO for PM_10_; after adjusting for SO_2_ or NO_2_ for PM_2.5_; and after adjusting for SO_2_, NO_2_, PM_10_, or PM_2.5_ for CO (Table 2). In addition, the associations between outdoor air pollutants and the PTB risk were robust when the maximum *df* of the TPSF varied (Table S3), at 53–313 weeks (Table S4), or based on the GAMM (Table S5).

**Figure 2.**
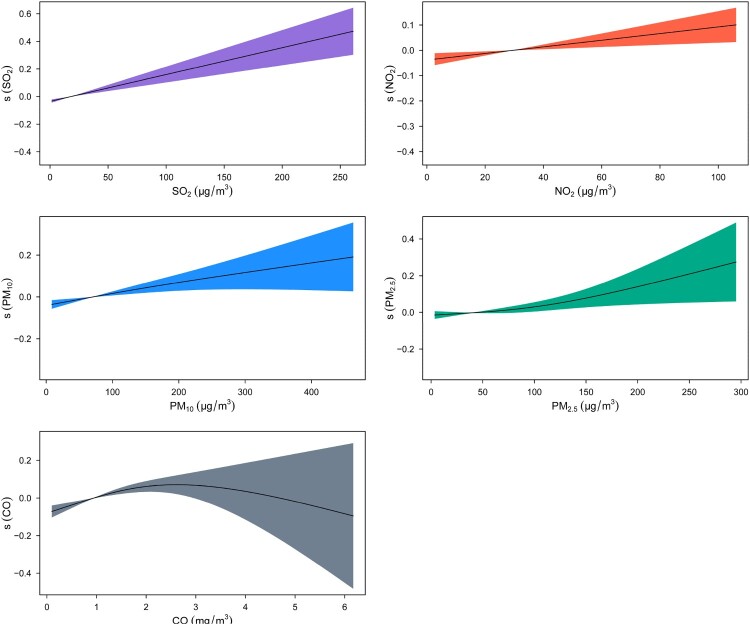
The concentration-response curves between ambient air pollutants and pulmonary tuberculosis risk. The x-axis represents the concentration of air pollutants, while the y-axis represents the contribution of the smooth term to the fitted values. We applied lag 0 week for SO_2_, lag 0 week for NO_2_, lag 8 weeks for PM_10_, lag 10 weeks for PM_2.5_, and lag 15 weeks for CO. The results were adjusted for the week, the number of cases in the previous week, the number of holidays in the week, season, city, average temperature, average wind speed, and average relative humidity at the same lag week.

The concentration-response curves between ambient air pollutants and the PTB risk were almost linear or approximately linear, except for CO (Figure 2). Therefore, we applied the piecewise linear function to re-evaluate its effect. The optimal cutoff value was identified as 2.3 mg/m^3^, corresponding to the minimum generalized cross-validation score (Table S6). For each 1-mg/m^3^ increase in CO, the PTB risk increased by 18.57% (95% CI: 10.82, 26.87) when CO was <2.3 mg/m^3^ and decreased by 22.51% (95% CI: −50.67, 21.75) when CO was ≥2.3 mg/m^3^ (Table S7).

## Discussion

In this study from different regions of China, we revealed a positive link between ambient SO_2_, NO_2_, PM_10_, PM_2.5_, and CO and PTB risk. This study goes beyond previous work to provide a rigorous, comprehensive assessment of the interplay between outdoor air pollution and PTB risk at the population level. To our knowledge, this is the largest and first multi-province, multi-city study to evaluate the impacts of ambient air pollutants on PTB risk.

A time-series analysis from Chengdu suggested that SO_2_ had adverse effects on tuberculosis [3]. Two spatiotemporal analyses in Seoul and Hubei also revealed a positive link between SO_2_ and tuberculosis [12,26]. The above studies support our findings. Nevertheless, no significant association was found between SO_2_ and tuberculosis in northern California or Lampang [2,27]. Northern California is economically developed and had low ambient SO_2_ concentrations during the study period (median: 3.43 μg/m^3^), while China is a developing country, and the median ambient SO_2_ concentration in our study was 13.57 μg/m^3^ [2]. Only a bivariate correlation analysis was conducted for Lampang to evaluate the effect of SO_2_ on the tuberculosis risk, but potential confounding factors were not considered [27]. These factors may partly explain the inconsistency.

We found that NO_2_ was positively associated with PTB at different lag weeks. Previous studies also reported that outdoor NO_2_ contributed to tuberculosis [2,3,28]. The sensitivity analyses suggested that this positive association was no longer significant when adjusting for SO_2_, possibly because SO_2_ and NO_2_ often coexist in the outdoor environment due to similar sources, and SO_2_ enhances the effect of NO_2_. The effect of NO_2_ exposure alone on PTB requires further study.

A study based on seven cities in Korea showed that PM_10_ had a lag effect on tuberculosis [29]. This finding was consistent with another study from Hong Kong [30]. Similarly, we found that PM_10_ had a relatively long-term effect on PTB, as the association became significant from lag 8 weeks. Nevertheless, a study from northern California indicated that PM_10_ played a protective role in the PTB risk [2]. Differences in socioeconomic level, PM_10_ composition, and concentration (median: 20.61 vs. 63.43 μg/m^3^) between the two study areas may be partly responsible for the opposite results.

A study in Beijing and Hong Kong reported that an increase in PM_2.5_ resulted in an increased risk of tuberculosis [18]. Similarly, we revealed a positive correlation between PM_2.5_ and PTB. The concentration-response curve between PM_2.5_ and PTB was approximately linear. However, the curve was relatively flat when the PM_2.5_ concentration was low and became steeper at higher concentrations, suggesting that high concentrations of PM_2.5_ had a greater impact on PTB. In addition, the influence of PM_2.5_ was slightly stronger than that of PM_10_, probably because, in contrast to PM_10_, PM_2.5_ has a smaller particle size and longer suspension time and can not only penetrate deep into the lungs but also enter the circulatory system through the respiratory barrier [31].

Studies from northern California and Hong Kong indicated that CO was positively correlated with tuberculosis [2,30]. We also observed a positive correlation between CO and PTB. Moreover, as the concentration-response curve between CO and PTB was nonlinear, we applied a piecewise linear function to re-estimate the effect. The results suggested that CO was positively correlated with PTB when the concentration was <2.3 mg/m^3^ and negatively linked to PTB when the concentration was ≥2.3 mg/m^3^, but the latter correlation was not significant. Compared to the piecewise linear function results, although our original results seemed to underestimate the effect of CO, they were still relatively reliable. The Q98 and Q99 of the CO concentration measurements were 2.04 and 2.33 mg/m^3^, respectively. In other words, approximately 99% of the measured concentrations were less than 2.3 mg/m^3^. The protective role of high concentrations of CO needs to be explored in future studies.

Although not statistically significant, we observed several reverse associations between air pollutants and PTB risk at several lag weeks. NO_2_ was negatively correlated with the PTB risk at shorter lag weeks, while PM_10_ and PM_2.5_ were negatively linked to the PTB risk at shorter and longer lag weeks, respectively. The negative correlations at shorter lag weeks may be related to two reasons. First, we utilized the diagnosis date instead of the onset date when counting the number of PTB patients. The onset date is inferred by clinicians based on the patients’ recollection, which is often inaccurate. The time from the onset of PTB to its diagnosis is usually several days. Second, PM_10_ and PM_2.5_ may have relatively long lag effects on the PTB risk, which was also observed in previous studies [18,32]. The negative associations at longer lag weeks may be attributed to two factors. One factor is that the lag effects of PM_10_ and PM_2.5_ on the PTB risk do not persist for long. The other factor is that a longer lag time results in more unknown confounding factors and a greater likelihood of distortion of the real correlations of PM_10_ and PM_2.5_ with the PTB risk. Moreover, we found that the relationship between outdoor air pollutants and PTB appeared to vary across populations and seasons, but these differences were statistically significant only between spring and winter for NO_2_, PM_10_, and PM_2.5_, which may be partly attributed to seasonal variations in air pollutant concentrations, sources, and PM compositions.

The mechanism for the role of outdoor air pollutants in PTB risk was beyond the scope of our study. However, several potential biological explanations may be primarily related to immune dysfunction. First, the respiratory tract can produce secretions to wrap *M.tb* and then clear it to defend against *M.tb* invasion. Exposure to outdoor air pollutants may weaken the mucociliary clearance of airway secretions, thus raising the possibility of *M.tb* infection [33]. Second, alveolar macrophages (AMs) are the first line of immune defense in the lungs, which can eliminate harmful bacteria. However, PM_10_ and PM_2.5_ exposure is likely to reduce the expression of phagocytosis-related receptor CD11b on the surface of AMs, impairing the ability of AMs to phagocytose *M.tb* [34]. Third, exposure to outdoor air pollutants decreased the expression of cytokines (TNF-α, IFN-γ, etc.) in peripheral blood mononuclear cells (PBMCs), suppressing the role of PBMCs in phagocytosing *M.tb* and controlling *M.tb* growth [35–37]. Fourth, PM_2.5_ is likely to cause inflammatory responses and intracellular oxidative stress, leading to the progression of granulomatous lesions in the lungs [31,38]. Large studies investigating tuberculosis risk, pollutant exposures, and immunological measures are needed to further address the mechanisms explaining the strong relationship we see in our research.

This study has several limitations. First, we utilized measurements from fixed stations to estimate individual exposure to outdoor air pollutants; however, exposure misclassification is unavoidable. Thus, more precise methods are needed to estimate individual exposure levels to air pollutants in future studies. For example, two studies from Changsha evaluated personal exposure to outdoor air pollutants using the inverse distance weighting method based on data from local air quality monitoring stations and the address of each participant [8,9]. Second, other confounders, such as comorbidities (diabetes, etc.) that impact the risk of tuberculosis and the quality of tuberculosis notification, were not included because they were not available in our database. Third, we extracted data on the diagnosis date of PTB patients, an inaccurate proxy of disease onset. This would lead to non-differential disease misclassification, likely biasing the associations we present here towards the null.

## Conclusions

In summary, we revealed that outdoor air pollutants were positively related to PTB risk. As many developing countries suffer from both a high tuberculosis burden and severe outdoor air pollution, reducing outdoor air pollutant concentrations might indirectly benefit tuberculosis control.

## Ethics approval

This study was approved by the ethics committee of Nanjing Medical University.

## Author contributions

Zhongqi Li, Qiao Liu, Wei Lu, and Jianming Wang designed the study. Zhongqi Li, Qiao Liu, Liang Chen, Liping Zhou, Wei Qi, Chaocai Wang, Yu Zhang, Bilin Tao, and Limei Zhu collected and organized the data. Zhongqi Li performed the analysis and visualization of the data. Qiao Liu and Bilin Tao verified the data. Zhongqi Li, Qiao Liu, Liang Chen, Liping Zhou, Wei Qi, and Chaocai Wang prepared the original manuscript. Leonardo Martinez, Wei Lu, and Jianming Wang guided the study and revised the original manuscript. Zhongqi Li, Qiao Liu, and Jianming Wang acquired the funding. All authors have reviewed and approved the manuscript.

## Supplementary Material

Supplementary Appendix.docx

## Data Availability

All data are available from the corresponding authors upon reasonable request.

## References

[1] WHO. Global tuberculosis report 2022. Geneva. Available from: https://www.who.int/publications/i/item/9789240061729

[2] Smith G S, Van Den Eeden SK, Garcia C, et al. Air pollution and pulmonary tuberculosis: a nested case-control study among members of a Northern California health plan. Environ Health Perspect. 2016;124(6):761–768. doi:10.1289/ehp.1408166 26859438 PMC4892908

[3] Zhu S, Xia L, Wu J, et al. Ambient air pollutants are associated with newly diagnosed tuberculosis: a time-series study in Chengdu, China. Sci Total Environ. 2018;631–632:47–55. doi:10.1016/j.scitotenv.2018.03.017 29524902

[4] WHO. Ambient Air pollution: a global assessment of exposure and burden of disease. Geneva. Available from: https://www.who.int/publications/i/item/9789241511353

[5] Liu C, Chen R, Sera F, et al. Ambient particulate air pollution and daily mortality in 652 cities. N Engl J Med. 2019;381(8):705–715. doi:10.1056/NEJMoa1817364 31433918 PMC7891185

[6] Tian Y, Liu H, Zhao Z, et al. Association between ambient air pollution and daily hospital admissions for ischemic stroke: a nationwide time-series analysis. PLoS Med. 2018;15(10):e1002668. doi:10.1371/journal.pmed.1002668 30286080 PMC6171821

[7] Young MT, Sandler DP, DeRoo LA, et al. Ambient air pollution exposure and incident adult asthma in a nationwide cohort of U. S. women. Am J Respir Crit Care Med. 2014;190(8):914–921. doi:10.1164/rccm.201403-0525OC 25172226 PMC4299575

[8] Jiang W, Lu C, Miao Y, et al. Outdoor particulate air pollution and indoor renovation associated with childhood pneumonia in China. Atmos Environ. 2018;174:76–81. doi:10.1016/j.atmosenv.2017.11.043

[9] Lu C, Yang W, Wang F, et al. Effects of intrauterine and post-natal exposure to air pollution on children’s pneumonia: key roles in different particulate matters exposure during critical time windows. J Hazard Mater. 2023;457:131837. doi:10.1016/j.jhazmat.2023.131837 37329598

[10] Saito Y, Azuma A, Kudo S, et al. Effects of diesel exhaust on murine alveolar macrophages and a macrophage cell line. Exp Lung Res. 2002;28(3):201–217. doi:10.1080/019021402753570509 11936774

[11] Saito Y, Azuma A, Kudo S, et al. Long-term inhalation of diesel exhaust affects cytokine expression in murine lung tissues: comparison between low- and high-dose diesel exhaust exposure. Exp Lung Res. 2002;28(6):493–506. doi:10.1080/01902140290096764 12217215

[12] Sohn M, Kim H, Sung H, et al. Association of social deprivation and outdoor air pollution with pulmonary tuberculosis in spatiotemporal analysis. Int J Environ Health Res. 2019;29(6):657–667. doi:10.1080/09603123.2019.1566522 30698032

[13] Liu Y, Cui L, Hou L, et al. Ambient Air pollution exposures and newly diagnosed pulmonary tuberculosis in Jinan, China: a time series study. Sci Rep. 2018;8(1):17411. doi:10.1038/s41598-018-35411-6 30479352 PMC6258663

[14] Chen R, Yin P, Wang L, et al. Association between ambient temperature and mortality risk and burden: time series study in 272 main Chinese cities. Br Med J. 2018;363:k4306. doi:10.1136/bmj.k4306 30381293 PMC6207921

[15] Liu C, Yin P, Chen R, et al. Ambient carbon monoxide and cardiovascular mortality: a nationwide time-series analysis in 272 cities in China. Lancet Planet Health. 2018;2(1):e12–e18. doi:10.1016/S2542-5196(17)30181-X 29615203

[16] Huang K, Ding K, Yang XJ, et al. Association between short-term exposure to ambient air pollutants and the risk of tuberculosis outpatient visits: a time-series study in Hefei, China. Environ Res. 2020;184:109343. doi:10.1016/j.envres.2020.109343 32192989

[17] Li Z, Liu Q, Zhan M, et al. Meteorological factors contribute to the risk of pulmonary tuberculosis: a multicenter study in eastern China. Sci Total Environ. 2021;793:148621. doi:10.1016/j.scitotenv.2021.148621 34328976

[18] You S, Tong YW, Neoh KG, et al. On the association between outdoor PM(2.5) concentration and the seasonality of tuberculosis for Beijing and Hong Kong. Environ Pollut. 2016;218:1170–1179. doi:10.1016/j.envpol.2016.08.071 27595179

[19] Li Z, Mao X, Liu Q, et al. Long-term effect of exposure to ambient air pollution on the risk of active tuberculosis. Int J Infect Dis. 2019;87:177–184. doi:10.1016/j.ijid.2019.07.027 31374344

[20] Zhu Y, Xie J, Huang F, et al. Association between short-term exposure to air pollution and COVID-19 infection: evidence from China. Sci Total Environ. 2020;727:138704. doi:10.1016/j.scitotenv.2020.138704 32315904 PMC7159846

[21] Amuakwa-Mensah F, Marbuah G, Mubanga M. Climate variability and infectious diseases nexus: evidence from Sweden. Infect Dis Model. 2017;2(2):203–217. 29928737 10.1016/j.idm.2017.03.003 PMC6002069

[22] Xie J, Zhu Y. Association between ambient temperature and COVID-19 infection in 122 cities from China. Sci Total Environ. 2020;724:138201. doi:10.1016/j.scitotenv.2020.138201 32408450 PMC7142675

[23] Zheng S, Zhu W, Wang M, et al. The effect of diurnal temperature range on blood pressure among 46,609 people in Northwestern China. Sci Total Environ. 2020;730:138987. doi:10.1016/j.scitotenv.2020.138987 32428804

[24] Chen Z, Liu P, Xia X, et al. Low ambient temperature exposure increases the risk of ischemic stroke by promoting platelet activation. Sci Total Environ. 2024;912:169235. doi:10.1016/j.scitotenv.2023.169235 38097078

[25] Lei R, Zhang L, Liu X, et al. Residential greenspace and blood lipids in an essential hypertension population: mediation through PM(2.5) and chemical constituents. Environ Res. 2024;240(Pt 1):117418. doi:10.1016/j.envres.2023.117418 37852460

[26] Liu F, Zhang Z, Chen H, et al. Associations of ambient air pollutants with regional pulmonary tuberculosis incidence in the central Chinese province of Hubei: a Bayesian spatial-temporal analysis. Environ Health. 2020;19(1):51. doi:10.1186/s12940-020-00604-y 32410699 PMC7226955

[27] Yasri S, Wiwanitkit V. Tuberculosis incidence in area with sulfur dioxide pollution: an observation. Med Gas Res. 2021;11(2):58–60. doi:10.4103/2045-9912.311490 33818444 PMC8130663

[28] Niu Z, Qi Y, Zhao P, et al. Short-term effects of ambient air pollution and meteorological factors on tuberculosis in semi-arid area, northwest China: a case study in Lanzhou. Environ Sci Pollut Res Int. 2021;28(48):69190–69199. doi:10.1007/s11356-021-15445-6 34291414

[29] Kim H, Yu S, Choi H. Effects of particulate air pollution on tuberculosis development in seven major cities of Korea from 2010 to 2016: methodological considerations involving long-term exposure and time lag. Epidemiol Health. 2020;42:e2020012. doi:10.4178/epih.e2020012 32164052 PMC7285441

[30] Xu M, Hu P, Chen R, et al. Association of long-term exposure to ambient air pollution with the number of tuberculosis cases notified: a time-series study in Hong Kong. Environ Sci Pollut Res Int. 2022;29(15):21621–21633. doi:10.1007/s11356-021-17082-5 34767173

[31] Feng S, Gao D, Liao F, et al. The health effects of ambient PM2.5 and potential mechanisms. Ecotoxicol Environ Saf. 2016;128:67–74. doi:10.1016/j.ecoenv.2016.01.030 26896893

[32] Lu JW, Mao JJ, Zhang RR, et al. Association between long-term exposure to ambient air pollutants and the risk of tuberculosis: a time-series study in Nantong, China. Heliyon. 2023;9(6):e17347. doi:10.1016/j.heliyon.2023.e17347 37441410 PMC10333459

[33] Houtmeyers E, Gosselink R, Gayan-Ramirez G, et al. Regulation of mucociliary clearance in health and disease. Eur Respir J. 1999;13(5):1177–1188. doi:10.1034/j.1399-3003.1999.13e39.x 10414423

[34] Ni L, Chuang CC, Zuo L. Fine particulate matter in acute exacerbation of COPD. Front Physiol. 2015;6:294. 26557095 10.3389/fphys.2015.00294 PMC4617054

[35] Ibironke O, Carranza C, Sarkar S, et al. Urban air pollution particulates suppress human T-cell responses to mycobacterium tuberculosis. Int J Environ Res Public Health. 2019;16(21):4112. doi:10.3390/ijerph16214112 31731429 PMC6862251

[36] Sarkar S, Rivas-Santiago CE, Ibironke OA, et al. Season and size of urban particulate matter differentially affect cytotoxicity and human immune responses to mycobacterium tuberculosis. PLoS One. 2019;14(7):e0219122. 31295271 10.1371/journal.pone.0219122 PMC6622489

[37] Sarkar S, Song Y, Sarkar S, et al. Suppression of the NF-κB pathway by diesel exhaust particles impairs human antimycobacterial immunity. J Immunol. 2012;188(6):2778–2793. doi:10.4049/jimmunol.1101380 22345648 PMC3293992

[38] Palanisamy GS, Kirk NM, Ackart DF, et al. Evidence for oxidative stress and defective antioxidant response in Guinea pigs with tuberculosis. PLoS One. 2011;6(10):e26254. doi:10.1371/journal.pone.0026254 22028843 PMC3196542

