# Supplementary material for: Ambient air pollution contributed to pulmonary tuberculosis in China
Source: Emerg Microbes Infect. 2024 Aug 29;13(1):2399275. doi: 10.1080/22221751.2024.2399275 (PMC11378674; doi:10.1080/22221751.2024.2399275)

**Supplementary Appendix**

Table S1. Weekly average air pollutant concentrations and meteorological factors at 67 study sites during the study period.

Table S2. The differences in the effects of air pollutants on pulmonary tuberculosis risk between subgroups (comparison based on Table 3).

Table S3. Percentage changes in pulmonary tuberculosis risk and their 95% confidence intervals for each 10-μg/m^3^ (1-mg/m^3^ for CO) increase in ambient air pollutant concentrations when the maximum degree of freedom of the thin plate spline function with different values.

Table S4. Percentage changes in pulmonary tuberculosis risk and their 95% confidence intervals for each 10-μg/m^3^ (1-mg/m^3^ for CO) increase in ambient air pollutant concentrations at 53-313 weeks.

Table S5. Percentage changes in pulmonary tuberculosis risk and their 95% confidence intervals for each 10-μg/m^3^ (1-mg/m^3^ for CO) increase in ambient air pollutant concentrations based on the GAM and the GAMM method.

Table S6. The generalized cross-validation score of the piecewise linear regression model of CO at lag 15 weeks with different cutoff values.

Table S7. Percentage changes in pulmonary tuberculosis risk and their 95% confidence intervals for each 1-mg/m^3^ increase in CO concentration at lag 15 weeks based on the piecewise linear regression model.

Figure S1. The Spearman correlation analysis on the correlations between weekly average air pollutant concentrations and meteorological factors at 67 study sites during the study period.

Table S1. Weekly average air pollutant concentrations and meteorological factors at 67 study sites during the study period.

| Variables | Minimum | Q25 | Median | Mean | Q75 | Maximum |
| --- | --- | --- | --- | --- | --- | --- |
| Air pollutant |  |  |  |  |  |  |
| SO_2_ (μg/m^3^) | 1.57 | 9.00 | 13.57 | 18.30 | 21.29 | 261.14 |
| NO_2_ (μg/m^3^) | 2.71 | 18.71 | 27.43 | 29.53 | 38.00 | 106.71 |
| PM_10_ (μg/m^3^) | 8.57 | 44.57 | 63.43 | 71.51 | 90.00 | 463.00 |
| PM_2.5_ (μg/m^3^) | 3.57 | 25.43 | 36.86 | 42.85 | 53.57 | 295.29 |
| CO (mg/m^3^) | 0.10 | 0.73 | 0.90 | 0.97 | 1.13 | 6.17 |
| Meteorological factor |  |  |  |  |  |  |
| Temperature (°C) | −21.80 | 8.66 | 18.01 | 15.95 | 24.76 | 35.13 |
| Wind speed (m/s) | 0.27 | 1.70 | 2.13 | 2.28 | 2.70 | 9.00 |
| Relative humidity (%) | 13.29 | 61.86 | 73.71 | 70.58 | 81.71 | 100.00 |

Table S2. The differences in the effects of air pollutants on pulmonary tuberculosis risk between subgroups (comparison based on Table 3)^*^.

| Subgroup | SO_2_ | NO_2_ | PM_10_ | PM_2.5_ | CO |
| --- | --- | --- | --- | --- | --- |
| Male vs. female | 0.212 | 0.707 | 0.820 | 1.191 | 0.938 |
| <60 years old vs. ≥60 years old | 0.001 | 0.676 | 0.164 | 0.106 | 0.291 |
| Spring vs. summer | 0.899 | 0.413 | 1.622 | 0.566 | 0.905 |
| Spring vs. autumn | 1.189 | 0.135 | 0.985 | 0.736 | 0.977 |
| Spring vs. winter | 1.675 | 2.688 | 3.665 | 2.663 | 1.143 |
| Summer vs. autumn | 0.102 | 0.299 | 0.217 | 0.209 | 1.605 |
| Summer vs. winter | 0.070 | 1.707 | 1.568 | 1.416 | 0.403 |
| Autumn vs. winter | 0.298 | 2.506 | 1.460 | 1.018 | 1.739 |

^*^: The difference between different gender groups or age groups was considered to be statistically significant if the corresponding value was >1.96, and the difference between different season groups was considered to be statistically significant after the Bonferroni correction if the corresponding value was >2.64.

Table S3. Percentage changes in pulmonary tuberculosis risk and their 95% confidence intervals for each 10-μg/m^3^ (1-mg/m^3^ for CO) increase in ambient air pollutant concentrations when the maximum degree of freedom of the thin plate spline function with different values^*,†^.

|  | Maximum degree of freedom | SO_2_ | NO_2_ | PM_10_ | PM_2.5_ | CO |
| --- | --- | --- | --- | --- | --- | --- |
| The thin plate spline function for week | 4 | 1.95 (1.24, 2.67) | 1.50 (0.62, 2.38) | 0.56 (0.26, 0.87) | 0.63 (0.20, 1.07) | 6.29 (3.44, 9.21) |
|  | 5 | 1.96 (1.25, 2.68) | 1.29 (0.42, 2.18) | 0.55 (0.24, 0.85) | 0.59 (0.16, 1.03) | 5.78 (2.94, 8.70) |
|  | 6 | 1.97 (1.26, 2.68) | 1.30 (0.43, 2.19) | 0.55 (0.24, 0.85) | 0.59 (0.16, 1.03) | 5.80 (2.96, 8.72) |
|  | 7 | 1.97 (1.26, 2.68) | 1.29 (0.42, 2.17) | 0.55 (0.25, 0.86) | 0.59 (0.16, 1.03) | 5.83 (2.99, 8.75) |
|  | 8 | 1.98 (1.27, 2.69) | 1.30 (0.43, 2.19) | 0.55 (0.25, 0.86) | 0.60 (0.17, 1.03) | 5.85 (3.01, 8.77) |
| The thin plate spline function for meteorological factors | 2 | 1.97 (1.26, 2.68) | 1.30 (0.43, 2.19) | 0.55 (0.24, 0.85) | 0.59 (0.16, 1.03) | 5.80 (2.96, 8.72) |
|  | 3 | 1.96 (1.25, 2.68) | 1.26 (0.39, 2.14) | 0.53 (0.22, 0.84) | 0.51 (0.08, 0.95) | 5.77 (2.93, 8.68) |
|  | 4 | 1.96 (1.25, 2.68) | 1.24 (0.37, 2.12) | 0.52 (0.21, 0.83) | 0.52 (0.09, 0.96) | 5.92 (3.08, 8.85) |
|  | 5 | 1.96 (1.25, 2.68) | 1.23 (0.36, 2.11) | 0.51 (0.21, 0.82) | 0.52 (0.09, 0.96) | 5.85 (3.00, 8.77) |
|  | 6 | 1.96 (1.25, 2.68) | 1.24 (0.37, 2.12) | 0.51 (0.21, 0.82) | 0.52 (0.09, 0.96) | 5.83 (2.99, 8.76) |

^*^: We applied lag 0 week for SO_2_, lag 0 week for NO_2_, lag 8 weeks for PM_10_, lag 10 weeks for PM_2.5_, and lag 15 weeks for CO. ^†^: Adjusted for the week, the number of cases in the previous week, the number of holidays in the week, season, city, average temperature, average wind speed, and average relative humidity at the same lag week.

Table S4. Percentage changes in pulmonary tuberculosis risk and their 95% confidence intervals for each 10-μg/m^3^ (1-mg/m^3^ for CO) increase in ambient air pollutant concentrations at 53-313 weeks^*,†^.

| Air pollutant | Percentage changes and their 95% confidence intervals |
| --- | --- |
| SO_2_ | 1.95 (1.10, 2.81) |
| NO_2_ | 1.46 (0.52, 2.41) |
| PM_10_ | 0.53 (0.20, 0.87) |
| PM_2.5_ | 0.61 (0.14, 1.08) |
| CO | 6.70 (3.59, 9.89) |

^*^: We applied lag 0 week for SO_2_, lag 0 week for NO_2_, lag 8 weeks for PM_10_, lag 10 weeks for PM_2.5_, and lag 15 weeks for CO. ^†^: Adjusted for the week, the number of cases in the previous week, the number of holidays in the week, season, city, average temperature, average wind speed, and average relative humidity at the same lag week.

Table S5. Percentage changes in pulmonary tuberculosis risk and their 95% confidence intervals for each 10-μg/m^3^ (1-mg/m^3^ for CO) increase in ambient air pollutant concentrations based on the GAM and the GAMM method^*^.

| Air pollutant | GAM^†^ | GAMM^‡^ |
| --- | --- | --- |
| SO_2_ | 1.97 (1.26, 2.68) | 1.97 (1.27, 2.69) |
| NO_2_ | 1.30 (0.43, 2.19) | 1.35 (0.48, 2.22) |
| PM_10_ | 0.55 (0.24, 0.85) | 0.55 (0.25, 0.86) |
| PM_2.5_ | 0.59 (0.16, 1.03) | 0.60 (0.17, 1.03) |
| CO | 5.80 (2.96, 8.72) | 5.84 (3.01, 8.75) |

Abbreviations: GAM=generalized additive model; GAMM=generalized additive mixed model. ^*^: We applied lag 0 week for SO_2_, lag 0 week for NO_2_, lag 8 weeks for PM_10_, lag 10 weeks for PM_2.5_, and lag 15 weeks for CO. ^†^: Adjusted for the week, the number of cases in the previous week, the number of holidays in the week, season, city, average temperature, average wind speed, and average relative humidity at the same lag week. ^‡^: Adjusted for the week, the number of cases in the previous week, the number of holidays in the week, season, average temperature, average wind speed, and average relative humidity at the same lag week.

Table S6. The generalized cross-validation score of the piecewise linear regression model of CO at lag 15 weeks with different cutoff values^*^.

| Cutoff value (mg/m^3^) | Generalized cross-validation score |
| --- | --- |
| 2.2 | 1.864117 |
| 2.3 | 1.864101 |
| 2.4 | 1.864135 |
| 2.5 | 1.864214 |
| 2.6 | 1.864331 |
| 2.7 | 1.864394 |

^*^: Adjusted for the week, the number of cases in the previous week, the number of holidays in the week, season, city, average temperature, average wind speed, and average relative humidity at lag 15 weeks.

Table S7. Percentage changes in pulmonary tuberculosis risk and their 95% confidence intervals for each 1-mg/m^3^ increase in CO concentration at lag 15 weeks based on the piecewise linear regression model^*^.

| CO at lag 15 weeks (mg/m^3^) | Percentage changes and their 95% confidence intervals |
| --- | --- |
| <2.3 | 18.57 (10.82, 26.87) |
| ≥2.3 | −22.51 (−50.67, 21.75) |

^*^: Adjusted for the week, the number of cases in the previous week, the number of holidays in the week, season, city, average temperature, average wind speed, and average relative humidity at lag 15 weeks.

Figure S1. The Spearman correlation analysis on the correlations between weekly average air pollutant concentrations and meteorological factors at 67 study sites during the study period.


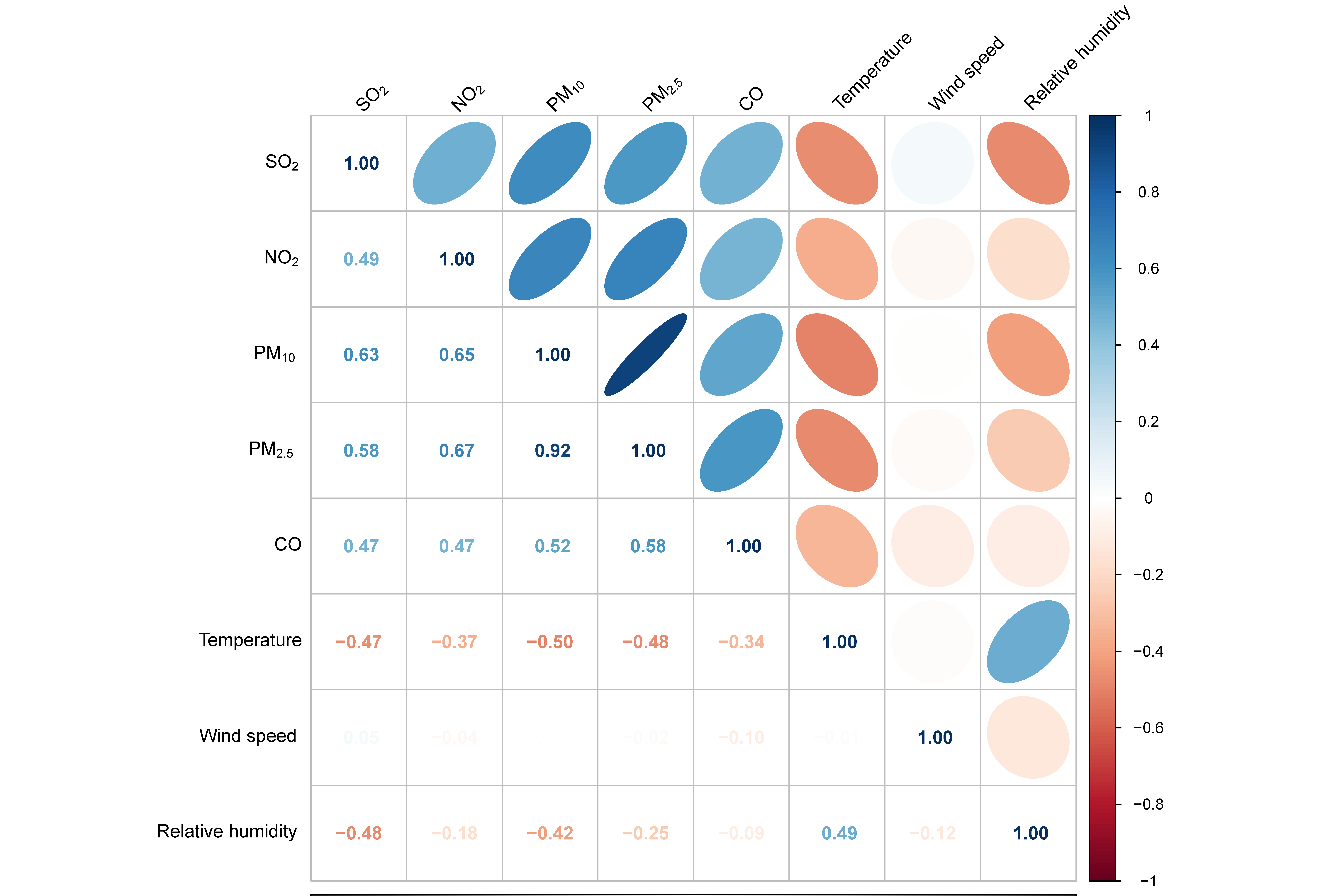

Supplement: Supplementary Appendix.docx [file TEMI_A_2399275_SM2126.docx]
